# Effect of the Blade-Coating Conditions on the Electrical and Optical Properties of Transparent Ag Nanowire Electrodes

**DOI:** 10.3390/mi14010114

**Published:** 2022-12-31

**Authors:** Hyungsub Yoon, Paolo Matteini, Byungil Hwang

**Affiliations:** 1 School of Integrative Engineering, Chung-Ang University, Seoul 06974, Republic of Korea; 2 Institute of Applied Physics “Nello Carrara”, National Research Council, 50019 Florence, Italy

**Keywords:** Ag nanowire, electrode, transparent, flexible, blading

## Abstract

Optimizing the coating conditions for a doctor blading system is important when seeking to improve the performance of Ag nanowire electrodes. In this study, the effect of the blading height and speed on the optical and electrical properties of Ag nanowire electrodes was investigated. Ag nanowires were first spread on a PET substrate using a doctor blade with differing heights at a fixed blading speed. An increase in the blading height resulted in the degradation of the optical transmittance and stronger haze due to the higher probability of Ag nanowire agglomeration arising from the greater wet thickness. When the blading speed was varied, the optical transmittance and haze were unaffected up until 20 mm/s, followed by minor degradation of the optical properties at blading speeds over 25 mm/s. The higher speeds hindered the spread of the Ag nanowire solution, which also increased the probability of Ag nanowire agglomeration. However, this degradation was less serious compared to that observed with a change in the blading height. Therefore, optimizing the blading height was confirmed to be the priority for the production of high-performance transparent Ag nanowire electrodes. Our study thus provides practical guidance for the fabrication of Ag nanowire electrodes using doctor blading systems.

## 1. Introduction

Transparent electrodes are an essential component for various state-of-the-art devices, including flexible displays, solar cells, electrochromic windows, and film-type heaters [1,2,3,4,5]. The material conventionally employed for the fabrication of transparent electrodes is indium tin oxide (ITO), but its use in flexible devices is limited due to the intrinsic brittleness and high processing costs of the resulting electrodes [6,7,8]. Therefore, a considerable volume of research has been devoted to seeking an alternative to ITO [9,10,11,12]. Of the various candidates proposed to date, Ag nanowires are considered one of the most promising for the fabrication of flexible transparent electrodes due to their low sheet resistance, high optical transmittance, and excellent mechanical flexibility [13,14,15,16]. 

In the electrode fabrication process, Ag nanowires are generally suspended in a liquid solvent such as deionized (DI) water, ethanol, or isopropyl alcohol (IPA), with the resulting solution coated on a transparent substrate using various coating or printing technologies such as doctor blading, spray coating, screen printing, or gravure printing [17,18]. Doctor blading is particularly widely used because of its simple process and low equipment costs [19,20,21,22]. A doctor blade is a blade sharpened to have a thin edge with a width below ~few microns. A sensitive jog controller enables control over the height of the doctor blade from the substrate on a microscale. During the coating process, an actuator pushes the doctor blade in the coating direction at a set speed, where inks ahead of the doctor blade spread in the direction of the doctor blade with the wet thickness corresponding to the height of the doctor blade. A typical coating method using a Mayer bar can produce scratches on the samples, while there are concerns that the other widely used spray coating methods have toxic chemicals that can be inhaled by persons near the process facility. Without such issues, doctor blading coating can deposit inks uniformly in a simple process through the precisely controlled movement of the doctor blade. Several types of blades such as lamella, bevel, or round blades are used for the doctor blade coating process, but the bevel blade is mostly used for low viscosity ink such as the Ag nanowire solution. In the doctor blading process, the Ag nanowire solution is dropped on one edge of the substrate and then spread across the entire area of the substrate via the movement of the doctor blade, with a specific gap maintained between the substrate and the blade tip. The quality of Ag nanowire electrodes produced in this manner is largely dependent on the doctor blading conditions, including the height of the blade and the blading speed. However, few studies have investigated the effect of the blading conditions on the optical and electrical properties of Ag nanowire electrodes [23,24,25]. For example, Krantz et al. prepared the Ag nanowire transparent electrode on glass substrates by using a doctor blading method, which achieved 90% optical transmittance and 10 ohm/sq using a blading speed of 5 mm/s [23]. However, the height of the doctor blade was not considered, which indicated that the effect of the height of the doctor blade on the electrode properties was not considered. In the work of Banica et al., Ag nanowire electrodes were deposited on polyethylene terephthalate (PET) substrates using a doctor blading method [24]. The height of the blade was fixed to have a ~30 μm gap from the PET substrates, which corresponded to ~180 μm for their system. The optical transmittance and the sheet resistance of the sample showing the best performance were ~87% and ~31 ohm/sq, respectively. However, the blading speed was not considered for controlling the optical and electrical performance of the Ag nanowire electrodes. Although those studies demonstrated Ag nanowire electrodes with high optical transmittance and low sheet resistance through the doctor blade method, the effects of blading speed and height were not considered in detail.

In this study, Ag nanowires suspended in IPA were spread on a PET substrate using a doctor blading system. The height of the blade and the blading speed were varied to confirm the influence of the doctor blading conditions on the Ag nanowire electrode performance in terms of the optical transmittance and haze. It was found that the optical properties were correlated with the sheet resistance. In addition, the figure of merit (FoM) was determined in order to discover the optimal blading conditions. These results provide practical guidance for the use of doctor blade systems in the fabrication of Ag nanowire electrodes.

## 2. Experimental

**Figure 1:**
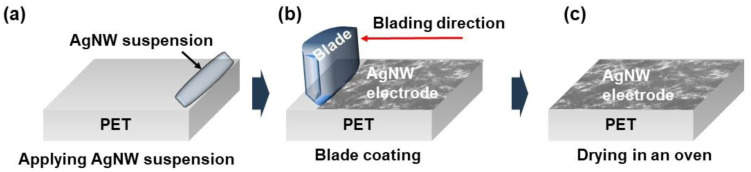
(**a**–**c**) Schematics of the coating process for the production of transparent Ag nanowire electrodes using a doctor blade system.

A Ag nanowire suspension in IPA (0.15 wt%) was purchased from Nanopyxis and used as received. Figure 1 presents a schematic of the coating process for Ag nanowire electrodes on a PET substrate using a doctor blade system. First, 2 mL of the Ag nanowire suspension was dropped in a line along one edge of the PET substrate. The doctor blade was positioned at the desired height and moved at a fixed speed, spreading the Ag nanowire solution across the entire area of the substrate along the direction of motion of the doctor blade. The height of the blade was controlled through the micro jog controller equipped within the system. The blading speed was controlled by pushing the speed in the system operated by an automatic actuator. The coated Ag nanowire electrodes were then dried in a convection oven at 80 °C for 20 min. The optical transmittance and haze were measured using a UV−vis spectrometer (haze-grad I, BYK-Gardner Instruments) following the ASTM D1003 standard (procedure A). The sheet resistance was measured using a four-point resistance measurement system (FPP-2400, Dasol Eng Co., Ltd.). The morphology of the Ag nanowire electrodes was also characterized using field-emission scanning electron microscopy (FE-SEM, SIGMA, Carl Zeiss).

## 3. Results and Discussion

**Figure 2:**
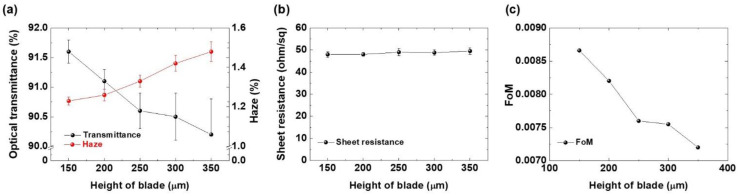
(**a**) Optical transmittance and haze, (**b**) sheet resistance, and (**c**) figure of merit (FoM) as a function of the blade height.

Figure 2a presents optical transmittance and haze values for the fabricated Ag nanowire electrodes as a function of the blade height. The thickness of the PET was 125 μm; thus, the blade height ranged from 150 μm to 350 μm. The doctor blade speed was fixed at 10 mm/s for this case. The optical transmittance of the Ag nanowire electrodes decreased as the height of the blade increased (91.6 ± 0.2% for a blade height of 150 μm and 90.2 ± 0.4% for a blade height of 350 μm). The haze exhibited a similar trend to that of the optical transmittance, with the Ag nanowire electrode coated with a blade height of 150 μm recorded at ~1.23 ± 0.03% and that coated with a blade height of 350 μm recorded at 1.48 ± 0.06%.

**Figure 3:**
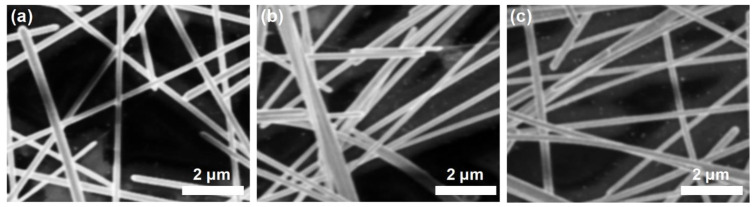
SEM images of Ag nanowire electrodes coated at a blade height of (**a**) 150 μm and (**b**) 350 μm with a blading speed of 10 mm/s. (**c**) SEM images of a Ag nanowire electrode coated at a blading speed of 30 mm/s with a blade height of 150 μm.

Figure 2b displays the sheet resistance as a function of the doctor blading height. There was no significant change in the sheet resistance, which may be because the same amount of Ag nanowire suspension volume was used for the tests. Figure 2c presents the FoM calculated using the optical transmittance and sheet resistance. The FOM is calculated based on the equation, FoM = T^10^/R_S_, where T and R_S_ are the optical transmittance and the sheet resistance, respectively. The highest FoM was obtained for the Ag nanowire electrode coated at a blade height of 150 μm. The greater blade height lowered the FoM due to the degradation of the optical transmittance. A smaller gap between the blade and substrate resulted in a uniform distribution of the Ag nanowires over the entire area of the PET substrate. On the other hand, when the gap was widened, the larger wet thickness of the Ag nanowire solution increased the probability of nanowire agglomerates due to the coffee ring effect [26,27]. In a coated layer, the solution at the edge evaporates faster than the inner region. Due to the capillary flow, the evaporated edge is replenished from the solution in the inner region, thereby forming high density layer of particles at the edge. In the case of the large wet thickness, the differences in the evaporation speed between the edge and the inner region become larger, which causes a more severe coffee ring phenomenon. In such a case, the differences in the density of the Ag nanowires between the edge and the inner region will be higher than those with smaller wet thickness, which might reduce the optical properties of the Ag nanowire electrodes. The non-uniformity caused by the agglomerated Ag nanowires would have degraded the optical transmittance and haze, as seen in Figure 2a. However, it was rather difficult to see the differences in agglomeration depending on the coating condition as shown in Figure 3. The Ag nanowire electrodes in the different coating conditions showed a similar distribution of Ag nanowires. There might be small differences in the degree of agglomeration, but differences in the optical properties were too small to be observed clearly in the global SEM images.

**Figure 4:**
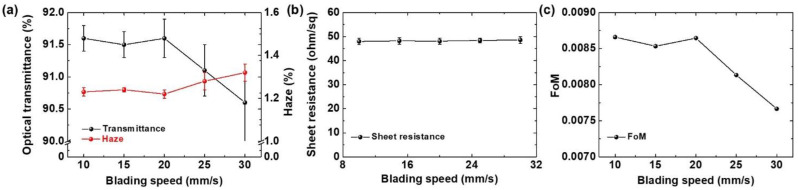
(**a**) Optical transmittance and haze, (**b**) sheet resistance, and (**c**) FoM as a function of the blading speed.

Doctor blading speed is another vital parameter that determines the properties of coated Ag nanowire electrodes. To confirm the effect of the doctor blading speed on Ag nanowire electrode performance, the Ag nanowire solution was spread on the PET substrate at different blading speeds with a fixed height of 150 μm. Figure 4a presents the optical transmittance and haze values of the Ag nanowire electrodes as a function of the blading speed. Up to a speed of 20 mm/s, the optical transmittance and haze demonstrated no significant change. Over 25 mm/s, however, the optical transmittance decreased, with a loss of 1% observed at a blading speed of 30 mm/s. The results for the haze were similar, with no significant change for blading speeds up to 20 mm/s. However, the haze increased from 1.23 ± 0.03% at a speed of 10 mm/s to 1.32 ± 0.05% at a speed of 30 mm/s.

There was no significant change in the sheet resistance as a function of the blading speed (Figure 4b). The FoM was similarly consistent at blading speeds below 20 mm/s and decreased at higher speeds (Figure 4c). The spread of the Ag nanowire solution was limited by the fast movement of the doctor blade, leading to the relatively larger agglomeration of Ag nanowires. With the fast movement of the doctor blade, the solution might be rather difficult to spread out across the substrate but instead just pushed out in the coating direction due to the surface tension. However, the deterioration in the optical properties of the Ag nanowire electrodes coated with higher blading speeds was less severe than observed for the increase in the blade height. There were also no significant differences in the SEM images of the Ag nanowire electrodes coated with blading speeds of 10 mm/s and 30 mm/s (Figure 3a,c, respectively). Therefore, optimizing the blade height for doctor blade coating systems should be the priority to obtain Ag nanowire electrodes with the optimal optical and electrical performance.

## 4. Conclusions

In this study, the effect of the doctor blading conditions on the optical and electrical properties of transparent Ag nanowire electrodes on a PET substrate was investigated. The Ag nanowire electrodes were coated on the PET substrate using a doctor blade at different blading heights and speeds. When the blading height was increased from 150 μm to 350 μm at a fixed speed of 10 mm/s, the optical transmittance of the Ag nanowire electrodes decreased and the haze increased. The sheet resistance exhibited no significant change with the blading height, while the FoM decreased as the blading height increased. A greater blade height resulted in an increase in the wet thickness, leading to Ag nanowire agglomerates, as confirmed by the SEM images, which resulted in the degradation of the optical properties of the Ag nanowire electrode.

The effect of the change on the blading speed was also investigated. Ag nanowires were coated at blading speeds from 10 mm/s to 30 mm/s. Up to 20 mm/s, there was no significant degradation in the optical and electrical properties. Over 25 mm/s, however, minor degradation was observed because the fast movement of the blade hindered the sufficient spread of the Ag nanowires, leading to their agglomeration. Therefore, the best optical and electrical properties of the Ag nanowire electrodes were achieved at a height of 150 nm and a speed of 10 mm/s. However, the change in the optical properties due to changes in the blading speed was less serious than for changes in the blading height. The blading height was confirmed to be the most important factor in fabricating high-performance Ag nanowire electrodes using a doctor blade system. The lower the blading height, the higher the electrode performance, which will be an interesting topic for our future study if we can obtain a new doctor blading machine that can control the height more precisely under 150 µm. Our results thus provide useful guidelines for the use of doctor blades in the coating of Ag nanowire electrodes.

## Data Availability

The datasets used and/or analyzed during the current study are available from the corresponding author on reasonable request.
